# Progress in Surgery

**Published:** 1895-06-15

**Authors:** 


### PROGRESS IN SURGERY.

### ANTISEPTICS.

In the surgery of the present the first point of success is the maintenance of an aseptic wound. This can only be secured by the practical application of an aseptic and antiseptic technique carefully carried out in the smallest details. The surgeon of to-day must know what absolute surgical cleanliness means, and have not only specially studied surgical anatomy, but have a clear and practical knowledge of the elements of bacteriology and of the pathological conditions which underlie suppuration in wounds and septic processes generally. These great advances in surgical methods are carefully set out in two recent works. The first, by Watson Cheyne[1] on "The Treatment of Wounds, Abscesses, and Ulcers" (July, 1894), deals with the present position of the Listerian method of treating wounds; the second, on "Aseptic Surgical Technique," by Hunter Robb,[2] with especial reference to gynecological operations. Cheyne's paper,[3] read before the West Somerset Branch of the British Medical Association, gives a concise view of the development of modern methods of wound treatment since the publication of his book on "Antiseptic Surgery." He does not, as a rule, boil his instruments, as he sees no advantage in it, nor does he think that the minute quantity of carbolic acid conveyed to the wound when they are disinfected by immersion in carbolic acid does any harm. Anything thoroughly soaked in 1 in 20 carbolic acid watery solution for 24 to 28 hours is absolutely disinfected.

"Where," says Cheyne, "we have to deal with unbroken skin at some distance from the mucous canals, failure of union by first intention is practically entirely due to imperfection in the methods employed by the surgeon, whether it be in his actual manipulations or in the asepticity of the materials which he supplies." Schimmelbusch's views on infection of wounds appear to be gaining ground in England as well as on the Continent. The second edition of his work on "The Aseptic Treatment of Wounds," which has been most ably translated by Dr. A. T. Rake,[4] contains a vast amount of information upon pathogenic micro-organisms, and should be in the hands of every surgeon. Henle, of Breslau,[5] lays stress upon the fact that Schimmelbusch's experiments were carried out with highly virulent organisms, pure cultures in large amounts, and that thus the natural conditions were not represented. Henle concludes that an antiseptic irrigation of a wound is a proper surgical procedure. Messner[6] experimented in the same direction to find how practicable it was to disinfect wounds which had been infected with fresh pus, or with pure cultures of pyogenic bacteria, by irrigation with 3 per cent. lysol or carbolic solution. Irrigation in nowise diminishes the vital energy of the tissues against pyogenic cocci, or predisposes the tissues to suppuration; in fact, the very opposite seems to be the case. In his "Notes on Aseptic Surgery," in the St. Bartholomew's Hospital Journal, C. B. Lockwood[7] gives an excellent and practical account of the micrococci and bacilli usually found in septic wounds. The properties of many of the bacteria are still uncertain; some are pathogenic, and others, perhaps, are non-pathogenic. Schleich[8] has recently employed preparations of blood serum as dressings for wounds and raw surfaces of skin and mucous membranes. Fresh bovine blood-serum is used, and mixed with 23 per cent. of zinc oxide, dried, and then reduced to powder. For foul ulcers and other septic conditions the powder may be obtained mixed with iodoform, boric acid, lysol, &c. Schleich uses a paste composed of the serum, wax, and zinc oxide, which mixes in any proportion with chrysarobin, lysol, ichthyol, resorcin, iodoform, &c., for eczema, burns, &c. The paste also mixes with mercury in every proportion. These albumen preparations serve as local stimulants to nutrition, and increase the circulation of leucocytes. Maffucci[9] has experimentally studied the resistance of embryonal tissue in contact with a certain virus compared with the tissue of the adult, and especially as regards the disposal of micro-organisms passing through the placenta and coming in contact with the tissues of the embryo. He concludes, that the living embryo can destroy, attenuate, or accumulate pathogenic microbes and develop them later in life. The antiseptic properties of iodoform have again been investigated by M. Stchegoleff[10] in Paris. Iodoform, while not attacking directly the staphylococcus, acts, nevertheless, on the toxine secreted by the microbe, so that under its influence the toxines of the staphylococcus are converted into non-poisonous iodised compounds. It is just the reverse in the case of the tubercle bacilli; when in contact with 5 per cent. of iodoform the tubercle bacilli die in forty-eight hours. W. A. Lane[11] has used sulphur, not only in the treatment of tuberculous disease, but also in disease resulting from the presence in the tissues of any form of organism. He believes that the advantage of the use of sulphur over sulphurous acid is analogous to that of iodoform over iodine. In the case of recent incisions,[12] sulphur should not be left in contact with the part which it is desired to sterilise for a longer period than twenty-four hours. When the circulation of the tissues operated on is impaired, it is frequently necessary to retain the sulphur for two, three or more days.

Although ichthyol is still extensively employed in many diseases of the skin, it appears to have but little antiseptic value. Its action on the growth of disease germs was found by Dunham[13] to be only slightly inhibitory. Its remarkable effects upon erysipelas, ulcers, and inflamed tissues have lately been fully confirmed. The utility of Unna's formula for arresting the progress of erysipelas—viz., icthyol, sp. ether ā ā ʒi., collodii ʒii., is endorsed by many surgeons.

Biniodide of mercury is strongly recommended by P. K. Bolshesolsky[14] as an antiseptic in obstetrical, as well as surgical practice. A 1 to 10,000 solution is quite sufficient for all purposes. He uses the biniodide for washing out the abdominal cavity in laparotomy, pleura in empyema, cerebral meninges in traumatic lesions, and synovial membranes in suppurative arthritis. Although he uses the solution very freely, he has never seen any untoward effects, and hence considers the drug "decidedly harmless." Klein and Parry Laws[15] have shown that phenylacetic and phenylpropionic acids have a stronger antiseptic action on pure cultures of anthrax bacilli than the higher acid, phenol, itself, phenylpropionic being stronger than phenylacetic. Laws[16] concludes that the phenyl-substituted fatty acids increase in antiseptic power with the increase of the molecular weight of the acid. Some time ago, Schinzinger suggested as a substitute for iodoform a new derivative of quinoline, viz., loretine. Through the iodine which this substance contains, and slowly gives off in a nascent state, it acts as an antiseptic. Loretine combining as an acid to form salts with the metallic oxides, Blum and Bärwald[17] have found that loretinate of bismuth is an especially valuable remedy, because it is endowed at the same time with the properties of iodine and of bismuth, being both antiseptic and astringent. It may, therefore, be employed advantageously in the case of ulcerated purulent surfaces, on which it exerts a very intense desiccating action. Trikresol[18] is a clear, colourless liquid three times as powerful as carbolic acid. It is cheap, makes a clear solution, does not corrode instruments, and neither makes the hands of the operator slippery, like lysol, nor numb, like carbolic acid. M. A. Trillat[19] adds to a former article upon the destructive influence of formol (methylic aldehyde) a short account of the process of disinfection by vapours of this substance, and gives the results of its antiseptic action.

[1] Treatment of Wounds, Abscesses, and Ulcers, Watson Cheyne, F.R.C.S., Y. J. Pentland, 1894, p. 197. [2] Aseptic Surgical Technique, with special reference to Gynæcological operations, Hunter Robb, M.D., Philadelphia, J. B. Lippincott, 1894, p. 264. [3] Lancet, Nov. 17th, 1894, p. 1,133. [4] The Aseptic Treatment of Wounds, by Dr. C. Schimmelbusch, translated by A. T. Rake, M.B., B.S., London, H. K. Lewis, 1894, p. 266. [5] Annals of Surgery, Oct., 1894, p. 471, and Verhand. der Deutsch Gesellschaft f. Chirurg., xxiii., Kongress, 1894. [6] Annals of Surgery, Oct., 1894, p. 472. [7] St. Bartholomew's Hospital Journal, Oct., 1894, p. 2. [8] The Practitioner, Jan., 1895, p. 82, and Therapeut. Monatschrift, Nov., 1894. [9] Epit. Brit. Med. Journ., Dec. 29th, 1894, and Annals of Surgery, Oct., 1894, Vol. XX., No. 4. [10] Lancet, Dec. 1st, 1894 [11] Brit. Med. Journ., Dec. 1st, 1894. [12] Lancet, Nov. 10th, 1894. [13] Med. Rec., Oct. 27th, 1894, and pamphlet, "The Physiology and Therapy of Ichthyol," Jan., 1895. [14] Brit. Med. Journ., Nov. 17th, 1894, and Proceedings of the Arkhangelsk Med. Soc., 1894. Vol ii., p. 19. [15] Epit. Brit. Med. Journ., Jan. 12th, 1895. [16] Journal of Physiology, Dec., 1894. [17] Med. Week, Sept. 28th, 1894. [18] Therap. Gazette, Oct. 15th, 1894, and Internat. Journ. of Surgery, Vol. VII., No. 3. [19] Journ. Bimensual, Oct. 26th, 1894.

